# Chestnut Wood Mud as a Source of Ellagic Acid for Dermo-Cosmetic Applications

**DOI:** 10.3390/antiox11091681

**Published:** 2022-08-28

**Authors:** Federica Moccia, Davide Liberti, Samuele Giovando, Carla Caddeo, Daria Maria Monti, Lucia Panzella, Alessandra Napolitano

**Affiliations:** 1 Department of Chemical Sciences, University of Naples “Federico II”, Via Cintia 4, I-80126 Naples, Italy; 2 Centro Ricerche per la Chimica Fine Srl for Silvateam Spa, Via Torre 7, I-12080 San Michele Mondovì, Italy; 3 Department of Scienze della Vita e dell’Ambiente, Sezione di Scienze del Farmaco, University of Cagliari, Via Ospedale 72, I-09124 Cagliari, Italy

**Keywords:** ellagic acid, chestnut wood, antioxidant, controlled release, transfersomes, HaCaT, 2,2-diphenyl-1-picrylhydrazyl (DPPH) assay, ferric reducing/antioxidant power (FRAP) assay, UVA, reactive oxygen species

## Abstract

Ellagic acid (EA) has long been recognized as a very active antioxidant, anti-inflammatory, and antimicrobial agent. However, its low bioavailability has often hampered its applications in health-related fields. Here, we report a phospholipid vesicle-based controlled release system for EA, involving the exploitation of chestnut wood mud (CWM), an industrial by-product from chestnut tannin production, as a largely available and low-cost source of this compound. Two kinds of CWM with different particle size distributions, indicated as CWM-A and CWM-B (<100 and 32 µm, respectively), containing 5 ± 1% *w*/*w* EA, were incorporated into transfersomes. The latter were small in size (~100 nm), homogeneously dispersed, and negatively charged. 2,2-Diphenyl-1-picrylhydrazyl (DPPH) and ferric reducing/antioxidant power (FRAP) assays indicated up to three-fold improvement in the antioxidant properties of CWM upon incorporation into transfersomes. The kinetics of EA released under simulated physiological conditions were evaluated by UV-Vis spectroscopy and HPLC analysis. The best results were obtained with CWM-B (100% of EA gradually released after 37 days at pH 7.4). A stepwise increase in the antioxidant properties of the released material was also observed. Cell-based experiments confirmed the efficacy of CWM-B transfersomes as antioxidant agents in contrasting photodamage.

## 1. Introduction

Ellagic acid (EA) is a phenolic compound naturally present in many red fruits and berries. Apart from being the main product of ellagitannin hydrolysis, it is endowed with remarkable biological properties, including antioxidant [1,2,3], anti-inflammatory [4], antimicrobial [5], antidiabetic [6], antiviral [7], antidegenerative [8], and anticancer activities [9]. In addition to systemic uses, topical applications of EA have been widely described [10]. Several studies have reported the potential use of EA for the prevention or treatment of skin disorders. For example, EA was found to be effective against skin tumors [11], contact dermatitis [12], or cutaneous leishmaniasis [13]. It can be used in wound bandaging [14], or as a photoprotective [15] and antiaging agent [16]. Furthermore, EA is considered a useful compound in the treatment of skin pigmentation disorders, such as hyperpigmentation, melasma, and other dyschromia [17].

Despite its remarkable properties, the wide application of EA is limited by its low permeability and low solubility in aqueous solvents. To overcome these drawbacks, several approaches have been proposed, involving modulation of EA solubility properties through encapsulation or chemical derivatization [18,19,20,21,22,23], and different type of formulations based on e.g., pectins [24,25], chitosan [25,26,27], chitin [28], zein [5], cellulose [29], cyclodextrins [30,31,32], poly(lactide-co-glycolide) (PLGA) [33], graphene oxide [34], alginate [35], and microalgae [36] have been designed for the controlled release of this compound.

In this context, liposomes (spherical vesicles composed of one or more bilayers formed by dispersion of phospholipids in aqueous medium) have been largely utilized as a drug delivery vehicle for administration of nutrients and pharmaceutical drugs in biomedical, food, and agricultural industries, and have also been exploited for enhancing the biological effects [37,38,39], improving skin permeation [40], and guaranteeing a sustained release [41] of EA. In particular, over the last few years, liposomes have been the target of reformulating studies aimed at producing vesicles capable of delivering active compounds to the deeper skin layers. A number of additives have been explored in combination with conventional components of liposomes, producing new classes of vesicles, such as transfersomes. Transfersomes are composed of phospholipids and an edge activator, which is a membrane-softening agent (e.g., Tween 80, Span 80, or sodium cholate) that makes the vesicle ultra-deformable and capable of penetrating the skin more efficiently than conventional liposomes [42,43,44,45].

In addition to the development of novel formulations to improve its bioavailability, another primary aim of the recent scientific research on EA is the discovery of sustainable, low cost and easily available sources of this compound, prompted by the global increasing demand for green products and processes. Among these sources, a prominent role is occupied by agri-food by-products such as pomegranate peel [46,47,48,49,50], although other ellagitannin-rich wastes have recently emerged as possible sources of EA. A noticeable example is represented by chestnut shell [51,52,53] as well as chestnut wood fiber, which is the residual exhausted material from chestnut tannin industrial production [54,55].

Within this scenario, we report herein the exploitation of chestnut wood mud (CWM) as an easily available source of EA for dermo-cosmetic applications upon incorporation into transfersomes. CWM is an industrial by-product of the chestnut tannin production, deriving from exhausted chestnut wood subjected to a natural fermentation process. The antioxidant properties of the samples were investigated by chemical assays and the protective effects on UVA-induced oxidative photodamage were evaluated on immortalized human keratinocytes (HaCaT). Finally, the controlled-release profile of EA under simulated physiological conditions was investigated by UV-Vis spectroscopy and HPLC.

## 2. Materials and Methods

### 2.1. Materials

CWM was provided by Silvateam (S. Michele Mondovì, Cuneo, Italy). CWM was first dried in an oven at 35 °C for one week, then ground in a common blender and finally passed through sieves to obtain two fractions with particle sizes lower than 100 and 32 µm, indicated as CWM-A and CWM-B, respectively.

Lipoid S75 (S75), a mixture of soybean phospholipids (70% phosphatidylcholine, 9% phosphatidylethanolamine and 3% lysophosphatidylcholine), triglycerides and fatty acids, was purchased from Lipoid GmbH (Ludwigshafen, Germany). Tween 80 (polysorbate 80, polyoxyethylene sorbitan monooleate; non-ionic hydrophilic surfactant, HLB 15) was supplied by Galeno (Carmignano, Prato, Italy).

2,2-Diphenyl-1-picrylhydrazyl (DPPH), iron(III) chloride (97%), phosphate buffer saline (PBS) 10×, 2,4,6-tris(2-pirydyl)-s-triazine (TPTZ) (≥98%), and (±)-6-hydroxy-2,5,7,8-tetramethylchromane-2-carboxylic acid (Trolox) (97%) were obtained from Sigma-Aldrich (Milan, Italy).

### 2.2. Methods

UV–Vis spectra were recorded on a Jasco (Lecco, Italy) V-730 Spectrophotometer.

HPLC analysis was performed with an Agilent (Cernusco sul Naviglio, Milan, Italy) instrument equipped with a UV-Vis detector; a Phenomenex (Castel Maggiore, Bologna, Italy) Sphereclone ODS column (250 × 4.60 mm, 5 µm) was used at a flow rate of 1.0 mL/min. A gradient elution using 0.1% formic acid in water (solvent A) and methanol (solvent B) was performed as follows: 5% B, 0–10 min; from 5 to 80% B, 10–57.5 min. The detection wavelength was set at 254 nm.

### 2.3. Preparation and Characterization of Transfersomes

CWM (A or B) was weighed in a glass vial along with S75; thereafter, Tween 80 and water were added (Table 1). To obtain the transfersomes, the dispersion was sonicated (5 s on and 2 s off, 10 cycles; 13 microns of probe amplitude) with an ultrasonic disintegrator (Soniprep 150 plus; MSE Crowley, London, UK).

For comparative purposes, empty transfersomes (i.e., those without CWM) were also prepared under the same conditions as CWM transfersomes (Table 1).

The mean diameter, polydispersity index, and zeta potential of the transfersomes were determined by dynamic and electrophoretic light scattering using a Zetasizer nano-ZS (Malvern Panalytical, Worcestershire, UK). Samples (*n* > 10) were diluted with water (1:100) and analyzed at 25 °C.

The above three parameters were monitored for 90 days to assess the long-term stability of the formulations.

### 2.4. Antioxidant Properties of CWM Samples

#### 2.4.1. DPPH Assay

CWM or CWM transfersomes (0.02–0.15 mg/mL final dose) (concentrations are referred to as CWM content in the formulations) were added to 3 mL of a 0.2 mM ethanolic solution of DPPH [56], and after 10 min under stirring at room temperature, the absorbance at 515 nm was measured. Experiments were run in triplicate.

#### 2.4.2. Ferric Reducing/Antioxidant Power (FRAP) Assay

CWM and CWM transfersomes were added (0.001–0.1 mg/mL final dose) (concentrations are referred to as CWM content in the formulations) to 3 mL of 0.3 M acetate buffer (pH 3.6) containing 1.7 mM FeCl_3_ and 0.83 mM TPTZ [57], and after 10 min of stirring at room temperature, the absorbance of the solutions at 593 nm was measured. Results were expressed as Trolox equivalents (eqs). Experiments were run in triplicate.

### 2.5. Release Experiments from CWM Transfersomes

Each CWM transfersome formulation (3 g) was placed in a dialysis membrane (MWCO 100–500 Da) and dialyzed against 30 mL of PBS 1×. The samples were kept at 37 °C in a water bath. Next, 0.5 mL of release medium was periodically withdrawn and replaced with an equal volume of corresponding fresh medium and analyzed using UV-Vis spectroscopy or HPLC. Each experiment was run in triplicate.

### 2.6. Antioxidant Properties of Released Fractions from CWM Transfersomes

Aliquots (150 µL) of the released fractions from CWM transfersomes were added to 2 mL of FRAP reagent prepared as described in Section 2.4.2. After 10 min under stirring, the mixtures were centrifuged (3 min at 5000 rpm) and the absorbance of the supernatants at 593 nm was measured.

### 2.7. Analysis of Cell Viability

Immortalized human keratinocytes (HaCaT, Innoprot, Derio, Spain) were cultured in 10% fetal bovine serum in Dulbecco’s Modified Eagle’s Medium, in the presence of 1% antibiotics and 2 mM l-glutamine, in a 5% CO_2_ humidified atmosphere at 37 °C. To verify the biocompatibility of each sample, cells were seeded in 96-well plates at a density of 2 × 10^3^/cm^2^ and 24 h after seeding. Cells were incubated in the presence of increasing concentration of EA (up to 10 µM) or transfersome samples (up to 25 µL/mL) for 24 and 48 h. At the end of incubation, cell viability was assessed by the 3-(4,5-dimethylthiazol-2-yl)-2,5-diphenyltetrazoliumbromide (MTT) assay. Cell survival was expressed as the percentage of viable cells in the presence of each sample and compared with control cells (represented by the average obtained between untreated cells and cells supplemented with the highest concentration of buffer). Each sample was tested in three independent analyses, each carried out in triplicate.

### 2.8. UVA Irradiation and Dichlorofluorescein Diacetate (DCFDA) Assay

The protective effect of each sample was measured by determining the intracellular reactive oxygen species (ROS) levels. A previously reported protocol [58] was followed, with some modifications. Briefly, HaCaT cells were preliminarily exposed for 2, 6, and 16 h to 10 µM EA to define the proper incubation time. After that, cells were incubated with the samples (10 µM EA or 25 µL/mL transfersomes, providing a 10 µM EA concentration) for 6 h in the absence or presence of 10 min UVA irradiation (100 J/cm^2^). At the end of the irradiation, H_2_-DCFDA was added to measure intracellular ROS level. Fluorescence intensity of the probe was measured at an emission wavelength of 525 nm and an excitation wavelength of 488 nm using a Perkin-Elmer (Milan, Italy) LS50 spectrofluorometer. Emission spectra were acquired at a scanning speed of 300 nm/min, with 5 slit widths for both excitation and emission. ROS production was expressed as percentage of DCF fluorescence intensity of the sample under test, compared to the untreated sample. Results are presented as mean of results obtained after three independent experiments (mean ± SD) and compared by one-way ANOVA according to Bonferroni’s method (post hoc) using Graphpad Prism for Windows, version 6.01.

## 3. Results and Discussion

### 3.1. Determination of the EA Content in CWM Samples

To gain information about the amount of EA contained in the two CWM samples, DMSO solutions of CWM-A and CWM-B were prepared and then analyzed using UV-Vis spectroscopy and HPLC after proper dilution in methanol. DMSO was chosen as the solvent based on its ability to dissolve a wide range of most polar and non-polar natural phenolic compounds, including EA [50,54,59].

**Figure 1:**
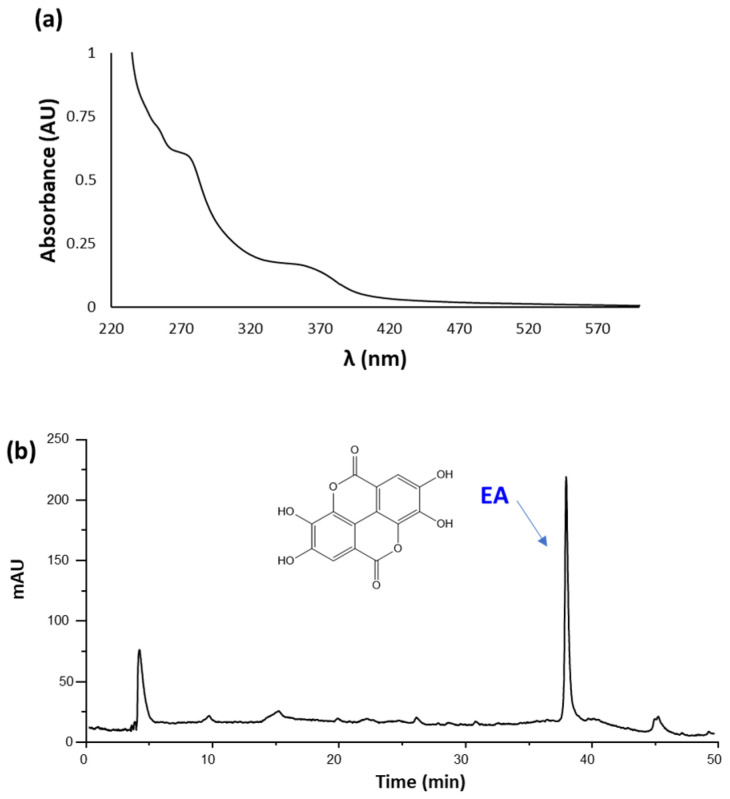
(**a**) UV-Vis spectrum (recorded at 0.02 mg/mL) and (**b**) HPLC profile (recorded at 1 mg/mL) of CWM-A.

As an example, the UV-Vis spectrum and elutographic profile of CWM-A are reported in Figure 1. The UV-Vis spectrum was characterized by absorption maxima at around 280 and 360 nm, as expected based on the presence of EA [60]. In agreement with this observation, HPLC analysis showed the presence of a single chromatographable compound eluted at ca. 38 min, identified as EA by comparison with an authentic standard. Quantitative analysis indicated content of EA of 5 ± 1% *w*/*w* for both CWM-A and CWM-B.

### 3.2. Incorporation of CWM Samples into Transfersomes

Transfersomes—that is, phospholipid vesicles modified with Tween 80 to promote skin penetration—containing CWM were produced and characterized in terms of size, homogeneity, and surface charge. To evaluate the CWM effect on the vesicles, the CWM transfersomes were compared with the empty transfersomes.

The light scattering results, as reported in Table 2, showed that the empty transfersomes had a mean diameter of 106 nm, and were homogeneously dispersed (polydispersity index 0.27) and highly negatively charged (−71 mV). CMW-A incorporation significantly increased the mean diameter of the vesicles, although they remained small (around 120 nm); the polydispersity index was unaltered, and the zeta potential value became less negative (Table 2), but it was still high enough to allow particle repulsion and prevent aggregation. On the other hand, CMW-B incorporation did not affect the vesicle size, nor the homogeneity of the dispersion, but produced less negative surface charge, as much as CMW-A.

The stability of the transfersome formulations was evaluated by monitoring the mean diameter, the polydispersity index, and the zeta potential during a 90-day storage period at 4 °C. No significant alterations (<10%) were detected.

The physicochemical characteristics of the herein described transfersomes are in line with those reported in literature for other EA-incorporating nanosystems [20]. As an example, Tween 80-coated chitosan-based nanoformulations exhibited an average hydrodynamic diameter of 155 nm and a PI of 0.37, although a lower ZP (−9.7 mV) compared to CWM transfersomes was determined. These nanoformulations led to a sustained release of EA (47% after 24 h) at pH 7.4 and exhibited more efficient anticancer effects in tumor-bearing mice compared to EA alone [27]. EA-loaded schizophyllan and chitin nanoparticles showed size distributions of 217.8 and 39.82 nm, and ZP of +27 and −9.14 mV, respectively. The chitin nanoparticles in particular led to a rapid release of EA (ca. 50%) after 8 h at pH 7.4, followed by a gradual release (up to 63%) that continued up to 50 h. MTT assay indicated that both nanoformulations effectively inhibited the growth of breast cancer cell lines, with IC_50_ values of 60 and 115 μg/mL, respectively [28]. Zein nanoparticles containing EA showed a mean size between 260 and 370 nm and a PI lower than 0.3. These formulations were found to be positively charged, with ZP ranging from +24 to + 37 mV, and showed inhibitory and bactericide activity against *S. aureus* and *P. aeruginosa* (MIC <72 μg/mL) [5]. Finally, poly(ε-caprolactone)-based EA nanoparticles formulated by applying various stabilizing agents exhibited average diameters ranging from 193 to 1252 nm, PI of 0.36–0.98 and ZP of −25–+62 mV. A fast release followed by a linear release period with a slower rate was observed at pH 7.4, with a cumulative release ranging from 25% to 48% after 8 days. These nanoparticles enhanced the cytotoxicity of EA up to 6.9-fold against colon adenocarcinoma cells, as well as the absorption extent of orally taken EA in rabbits [61].

### 3.3. Antioxidant Properties of CWM Transfersomes

The antioxidant properties of the CWM transfersomes were initially investigated with respect to the starting CWM samples by widely used chemical assays; that is, the DPPH and FRAP assays. Standard EA was also tested for comparison. The results are shown in Table 3. Both CWM-A and CWM-B exhibited antioxidant properties in line with what was expected based on a 5% *w*/*w* EA content. Notably, incorporation into transfersomes induced an about 2.5-fold decrease in the EC_50_ values determined in the DPPH assay for the CWM samples, and an even higher improvement in the reducing properties was observed in the FRAP assay. Since empty transfersomes were not found to exhibit significant antioxidant properties, these results clearly suggest a larger availability of the antioxidant compound EA following incorporation into the vesicles.

### 3.4. Release of EA from CWM Transfersomes and Antioxidant Properties of the Released Fractions under Simulated Physiological Conditions

**Figure 2:**
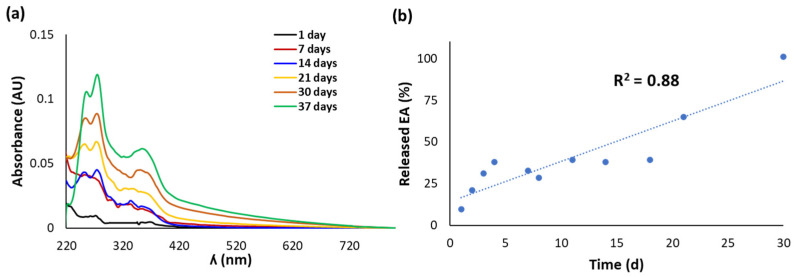
(**a**) UV-Vis spectra of fractions released over time from CWM-B transfersomes in PBS at 37 °C. (**b**) Kinetics of release of EA, determined by HPLC analysis. Reported are the mean values of at least three experiments (SD ≤ 10%).

The release of EA from the CWM transfersomes in PBS at 37 °C was followed by UV-Vis spectroscopy and HPLC over 5 weeks. No significant release of EA was observed in the case of CWM-A, probably as a result of the higher particle size of the sample, whereas very promising results were obtained with the CWM-B transfersomes. Indeed, the UV-Vis spectra of the released fractions from the latter exhibited absorption maxima at ca. 280 and 360 nm, which linearly increased over time (Figure 2a). HPLC analysis confirmed a controlled release of EA, which was complete after 30 days, reaching a concentration of ca. 56 µM (Figure 2b).

**Figure 3:**
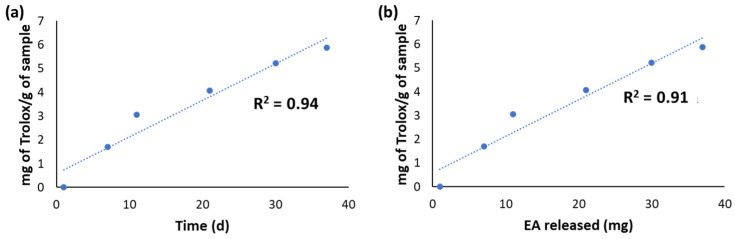
(**a**) Results of the FRAP assay on the fractions released over time from CWM-B transfersomes in PBS at 37 °C. (**b**) Correlation between the Fe^3+^-reducing properties of the released fractions and the amount of released EA.

The released fractions from CWM-B transfersomes were also evaluated for their antioxidant properties by chemical assays. Actually, it was not possible to perform the DPPH assay due to interference of the released material with the assay medium. On the other hand, the reducing properties evaluated by the FRAP assay (Figure 3a) linearly increased over time on account of the progressive release of EA from the transfersomes. A good linear correlation (R^2^ = 0.91) of the antioxidant properties with the amount of total released EA was indeed observed (Figure 3b).

### 3.5. Cell Viability of CWM Transfersomes

Based on the encouraging results of the release experiments, and with the aim of further probing the potential of CWM transfersomes for dermo-cosmetic applications, in subsequent experiments the sample biocompatibility was evaluated on HaCaT, since these cells are normally present in the outermost layer of the skin. EA was also tested for comparison in a range of concentrations corresponding to those provided by the CWM transfersome samples. MTT assay (not shown) showed that both EA (up to 10 µM) and the transfersomes (up to 25 µL/mL) were biocompatible under all the experimental conditions.

### 3.6. Protective Effect of CWM Transfersomes on Photoinduced Oxidative Stress

**Figure 4:**
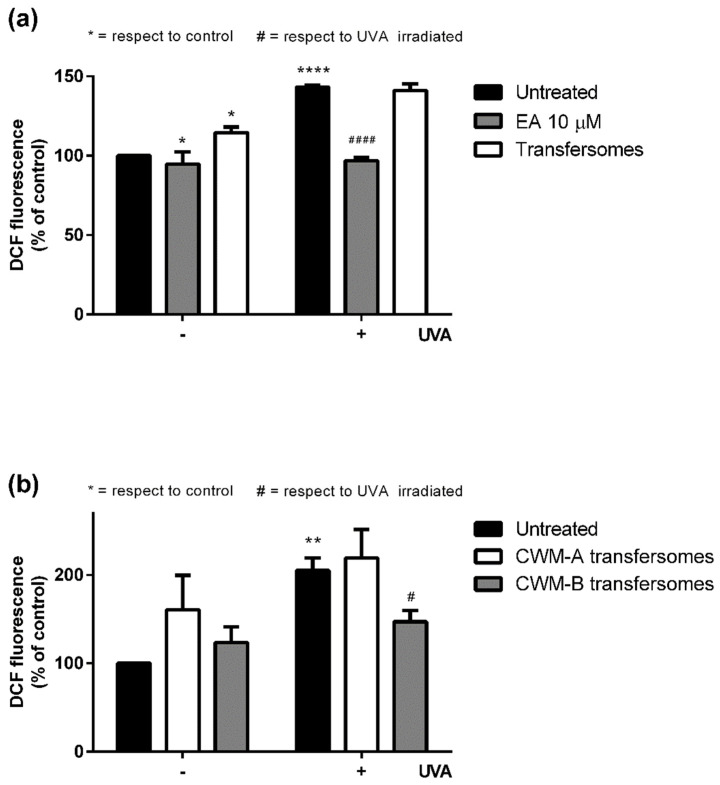
Protective effects of EA and transfersome samples on UVA-stressed HaCaT cells. Intracellular ROS levels were determined by DCFDA assay. Cells were preincubated with (**a**) 10 μM EA (gray bars) or 25 µL/mL empty transfersomes (white bars), or (**b**) 25 µL/mL of CWM-A (white bars) or CWM-B (gray bars) transfersomes (both providing a 10 µM EA concentration). Black bars refer to untreated cells in the absence (-) or in the presence (+) of UVA stress. Values are expressed as percentage with respect to untreated cells. Data shown are means ± SD of three independent experiment. *, # indicate *p* < 0.05; ** indicates *p* < 0.01; ****, #### indicate *p* < 0.001 with respect to control cells and UVA treated cells, respectively.

The antioxidant cytoprotective properties of CWM-A and CWM-B transfersomes were evaluated on UVA-irradiated HaCaT. Preliminary experiments (data not shown) were performed to define the optimal time (2, 6, or 16 h) for cell preincubation with 10 μM EA (corresponding to a non-cytotoxic concentration of 25 µL/mL CWM transfersomes), and 6 h incubation was chosen for further experiments. As shown in Figure 4, UVA irradiation induced a significant increase in intracellular ROS levels (150–200%) with respect to untreated cells. When cells were pretreated with 10 μM EA (Figure 4a, gray bars) prior to UVA exposure, a significant lowering of intracellular ROS levels was observed. As expected, empty transfersomes did not exert any protective effect against oxidative stress. Interestingly, when cells were treated with 25 µL/mL CWM-B transfersomes (providing an EA concentration of 10 µM) (Figure 4b, gray bars) prior to UVA exposure, a significant reduction (*p* ≤ 0.05) in intracellular ROS levels, compared to untreated UVA-exposed cells, was observed. On the other hand, CWM-A transfersomes (Figure 4b, white bars) were unable to protect cells from UVA-induced oxidative stress injury. Thus, these results, combined with those from the release experiments, suggest that the particle size of the CWM incorporated into transfersomes is fundamental to allow EA to be active as an antioxidant in cellular models.

## 4. Conclusions

In conclusion, the present work reports the efficacy of transfersomes as carriers for the controlled release of the biologically active compound EA from CWM, an industrial by-product deriving from tannin extraction. The incorporation into the transfersomes induced a significant improvement of the antioxidant properties of CWM, likely as a result of the larger availability of EA. Moreover, the transfersomal CWM-B was found to be able to decrease ROS production in UVA-irradiated keratinocytes and to provide a complete and controlled release of EA in pseudophysiological conditions at pH 7.4, a result of interest in dermo-cosmetic applications. For example, open wounds are characterized by a neutral or alkaline pH ranging from 6.5 to 8.5, whereas chronic wounds exhibit a pH in the range of 7.5–8.5 [62,63]. All together, these results highlight nanoformulated CWM of proper particle size as an easily accessible and biocompatible material that could warrant a sustained release of the water-insoluble bioactive EA under physiologically relevant conditions; for example for the treatment and protection of damaged skin.

## Figures and Tables

**Table 1:** Composition of the transfersome formulations.

Formulation	S75	CWM	Tween 80	H_2_O
Empty transfersomes	120 mg	-	0.05 mL	0.95 mL
CWM-A transfersomes	120 mg	2 mg	0.05 mL	0.95 mL
CWM-B transfersomes	120 mg	2 mg	0.05 mL	0.95 mL

**Table 2:** Characteristics of empty and CWM transfersomes: mean diameter (MD), polydispersity index (PI), and zeta potential (ZP). Each value represents the mean ± SD (*n* > 10). * values statistically different (*p* < 0.05) with respect to empty transfersomes.

Formulation	MD (nm)	PI	ZP (mV)
Empty transfersomes	106 ± 3.1	0.27 ± 0.01	−71 ± 5.8
CWM-A transfersomes	* 121 ± 7.8	0.27 ± 0.01	* −56 ± 5.7
CWM-B transfersomes	105 ± 2.9	0.27 ± 0.03	* −58 ± 9.4

**Table 3:** Antioxidant properties of CWM samples. Reported are the mean ± SD values of at least three experiments. Data for CWM transfersomes have been normalized based on the CWM content in the formulation.

	DPPH Assay EC_50_ (mg/mL)	FRAP Assay (mg of Trolox/mg of Sample)
CWM-A transfersomes	0.0389 ± 0.0005	0.36 ± 0.06
CWM-B transfersomes	0.0375 ± 0.0004	0.39 ± 0.04
Empty transfersomes	-	0.00015 ± 0.00002
CWM-A	0.103 ± 0.001	0.047 ± 0.002
CWM-B	0.106 ± 0.001	0.050 ± 0.001
EA	0.0051 ± 0.0004	1.04 ± 0.02

## Data Availability

The data are contained within the article.
